# Elucidation of DNA Repair Function of PfBlm and Potentiation of Artemisinin Action by a Small-Molecule Inhibitor of RecQ Helicase

**DOI:** 10.1128/mSphere.00956-20

**Published:** 2020-11-25

**Authors:** Niranjan Suthram, Siladitya Padhi, Payal Jha, Sunanda Bhattacharyya, Gopalakrishnan Bulusu, Arijit Roy, Mrinal Kanti Bhattacharyya

**Affiliations:** a Department of Biochemistry, School of Life Sciences, University of Hyderabad, Hyderabad, India; b TCS Innovation Labs-Hyderabad (Life Sciences Division), Tata Consultancy Services Limited, Hyderabad, India; c Department of Biotechnology and Bioinformatics, School of Life Sciences, University of Hyderabad, Hyderabad, India; The Hebrew University of Jerusalem

**Keywords:** PfBlm, PfWrn, DNA repair, homologous recombination, *Plasmodium falciparum*, yeast complementation, molecular docking

## Abstract

Artemisinin (ART)-based combination therapies are recommended as first- and second-line treatments for Plasmodium falciparum malaria. Here, we investigated the impact of the RecQ inhibitor ML216 on the repair of ART-mediated damage in the genome of P. falciparum. *PfBLM* and *PfWRN* were identified as members of the RecQ helicase family in P. falciparum. However, the role of these RecQ helicases in DNA double-strand break (DSB) repair in this parasite has not been explored. Here, we provide several lines of evidence to establish the involvement of PfBlm in DSB repair in P. falciparum. First, we demonstrate that PfBlm interacts with two well-characterized DSB repair proteins of this parasite, namely, PfRad51 and PfalMre11. Second, we found that *PfBLM* expression was upregulated in response to DNA-damaging agents. Third, through yeast complementation studies, we demonstrated that *PfBLM* could complement the DNA damage sensitivity of a Δ*sgs1* mutant of Saccharomyces cerevisiae, in contrast to the helicase-dead mutant *PfblmK83R*. Finally, we observe that the overexpression of *PfBLM* induces resistance to DNA-damaging agents and offers a survival advantage to the parasites. Most importantly, we found that the RecQ inhibitor ML216 inhibits the repair of DSBs and thereby renders parasites more sensitive to ART. Such synergism between ART and ML216 actions was observed for both drug-sensitive and multidrug-resistant strains of P. falciparum. Taken together, these findings establish the implications of PfBlm in the *Plasmodium* DSB repair pathway and provide insights into the antiparasitic activity of the ART-ML216 combination.

**IMPORTANCE** Malaria continues to be a serious threat to humankind not only because of the morbidity and mortality associated with the disease but also due to the huge economic burden that it imparts. Resistance to all available drugs and the unavailability of an effective vaccine cry for an urgent discovery of newer drug targets. Here, we uncovered a role of the PfBlm helicase in *Plasmodium* DNA double-strand break repair and established that the parasitic DNA repair mechanism can be targeted to curb malaria. The small-molecule inhibitor of PfBlm tested in this study acts synergistically with two first-line malaria drugs, artemisinin (ART) and chloroquine, in both drug-sensitive and multidrug-resistant strains of P. falciparum, thus qualifying this chemical as a potential partner in ART-based combination therapy. Additionally, the identification of this new specific inhibitor of the *Plasmodium* homologous recombination (HR) mechanism will now allow us to investigate the role of HR in *Plasmodium* biology.

## Abstract (precis)

Malaria continues to be a serious threat to humankind not only because of the morbidity and mortality associated with the disease but also due to the huge economic burden that it imparts. Resistance to all available drugs and the unavailability of an effective vaccine cry for an urgent discovery of newer drug targets.

## INTRODUCTION

Malaria is a deadly disease that accounts for major deaths across the world. Plasmodium falciparum is the most virulent among the five species of *Plasmodium* known to cause malarial pathology. Moreover, complete eradication of malaria has been difficult because of the continuous emergence of resistance to available drugs (1) and unsuccessful vaccine development efforts. Consequently, there is a pressing need to explore the pathways implicated in pathogenicity to ensure better understanding and targeted drug discovery.

The DNA double-strand break (DSB) repair pathway is one such reliable pathway in unicellular organisms since a single unrepaired DSB leads to the death of the organism (2). P. falciparum, being a unicellular organism, becomes vulnerable if not repaired. Previous reports have shown homologous recombination (HR) to be one of the primary pathways for DSB repair in *Plasmodium* (3). Although there is a possibility of an alternative end-joining pathway, it is a rare event (4). Key proteins of the HR pathway, such as PfRad51 and PfalMre1, have been identified and characterized (5, 6). Since these genes share high homology with their human counterparts, it is reasonable that we explore less-conserved genes.

RecQ family DNA helicases are considered the gatekeepers of the genome owing to their predominant roles in various DNA metabolic processes (7). In humans, five RecQ helicases have been characterized; however, in yeast (*SGS1*) and bacteria (RecQ), only one RecQ helicase has been identified. Bloom’s syndrome helicase (*BLM*) is one such RecQ helicase among five RecQ helicases in humans, known for its pro- and antirecombinogenic activities to maintain genome stability (8). BLM helicase exhibits its prorecombinogenic activity at various steps of the HR pathway. The first step in HR involves the resectioning of the 5′ terminus of the DSB to produce 3′-single-stranded DNA (ssDNA) overhangs, mediated by either DNA2 or EXO1. The helicase activity of BLM is essential for the DNA2-mediated resectioning pathway (9), where it serves a helicase-independent stimulatory function in the Exo1-mediated pathway (10). Among all other RecQ helicases, the ability to stimulate resection is specific for BLM (9, 10). Previous research has shown that the SUMOylation of BLM facilitates the enrichment of the key HR protein Rad51 at the site of DNA damage (11). BLM stimulates the strand exchange activity of Rad51 when it is in the active ATP-bound form instead of the inactive ADP-bound form (12). BLM, along with TopoIII and RmiI, forms the dissolvasome complex to resolve double Holliday junctions, which results in the formation of noncrossover products, and this process is critical in somatic cells (13).

In *Plasmodium*, only two RecQ helicases, *PfBLM* and *PfWRN*, have been identified (14–16). Apart from the limited sequence similarity of their helicase domains, PfBlm and PfWrn are very divergent from their human orthologs (22.5% and 23.7% sequence similarity, respectively). Nonetheless, it was shown with purified PfBlm and PfWrn proteins that their helicase activities are conserved. Both proteins are found to be expressed during all intraerythrocytic stages of *Plasmodium* (15, 16). Recent studies have shown that PfBlm is essential for maintaining the clonal expression of *var* genes, and the rate of recombination at the *var* locus was unchanged in a Δ*Pfblm* strain (17, 18). Owing to the fact that *Plasmodium* solely relies on HR to repair DSBs, it is of the utmost importance to explore the functional roles of these RecQ proteins of the parasite during DSB repair. In this study, we implicate PfBlm in *Plasmodium* DSB repair. We demonstrate that a RecQ helicase inhibitor abrogates the repair of DNA damage. Finally, we provide compelling evidence that the synergistic interaction between the RecQ inhibitor and the DNA-damaging agent artemisinin (ART) holds true in both drug-sensitive and multidrug-resistant parasites.

## RESULTS

### *PfBLM* expression is maximal at the mitotically active schizont stage.

**FIG 1:**
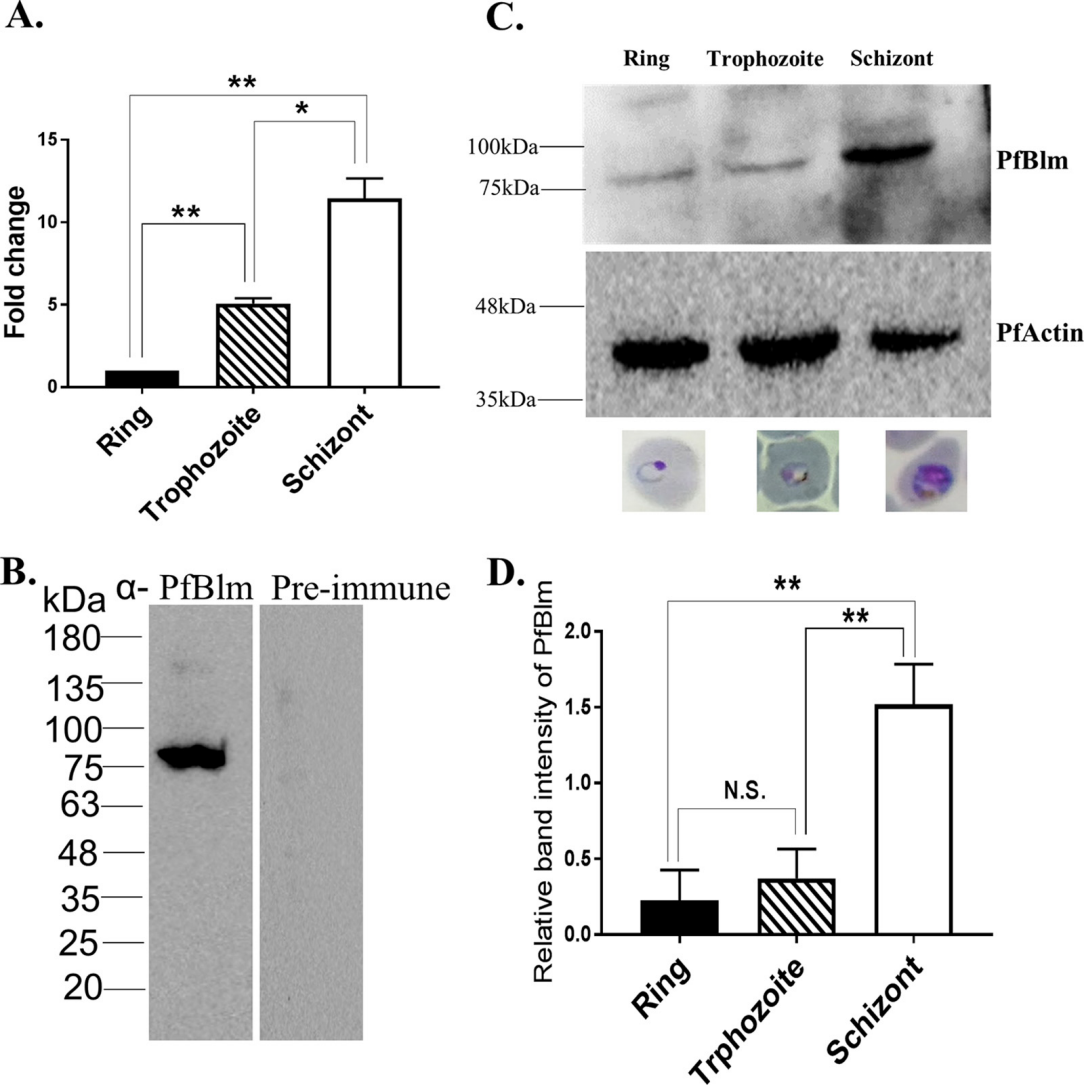
*PfBLM* expression is maximal at the mitotically active schizont stage. (A) Relative abundances of *PfBLM* transcripts during the ring (12 h postinvasion [hpi]), trophozoite (24 hpi), and schizont (38 hpi) stages quantified by real-time RT-PCR analysis. Data were normalized against *PfARP*. The mean values ± standard deviations (SD) from three independent experiments are plotted. (B) Specificity of anti-PfBlm antibody versus the preimmune sera. (C) Stage-specific expression of the PfBlm protein. PfActin was used as the loading control. The position of molecular markers is indicated on the left. Representative microscopic images of different blood stages are shown below the blot. (D) Quantification of Western blot data from three independent experiments. Data were normalized against loading control PfActin. Each bar represents mean density ± SD (*n* = 3). The *P* value was calculated using the two-tailed *t* test (* means a *P* value of <0.05, ** means a *P* value of <0.01, and N.S. means not significant).

To check the expression of *PfBLM* during blood stages of P. falciparum, we performed real-time reverse transcription-PCR (RT-PCR) and Western blotting. Previous reports have shown that *HsBLM* and *ScSGS1* expression levels are at their peak during the S phase of the cell cycle (19, 20). Since intraerythrocytic developmental stages of *Plasmodium* follow the pattern of the regular cell cycle, we sought to investigate its expression level during different blood stages. To this end, we isolated RNA and protein from synchronous parasites at the ring, trophozoite, and schizont stages to perform real-time RT-PCR and Western blotting. At the RNA level, the expression of *PfBLM* was found to be high during the schizont and trophozoite stages compared to the ring stage. *PfARP* (asparagine-rich protein) was used as a loading control. By real-time RT-PCR, we observed 5- and 11-fold upregulations of *PfBLM* in the trophozoite and schizont stages compared to the ring stage (Fig. 1A). These findings are in good agreement with several high-throughput transcriptome data sets available in the PlasmoDB database (21, 22). For Western blot experiments, we used an antipeptide antibody that recognized a specific band of 80 kDa corresponding to PfBlm (Fig. 1B). The schizont-stage-specific abundant expression of the PfBlm protein corroborated well with the mRNA expression data (Fig. 1C and D). PfActin was used as a loading control for the Western blot experiments. As the schizont stage is associated with replication and HR activities, our results suggest the likely involvement of PfBlm in these two conserved processes.

### DNA damage-induced upregulation of *PfBLM* during intraerythrocytic developmental stages.

**FIG 2:**
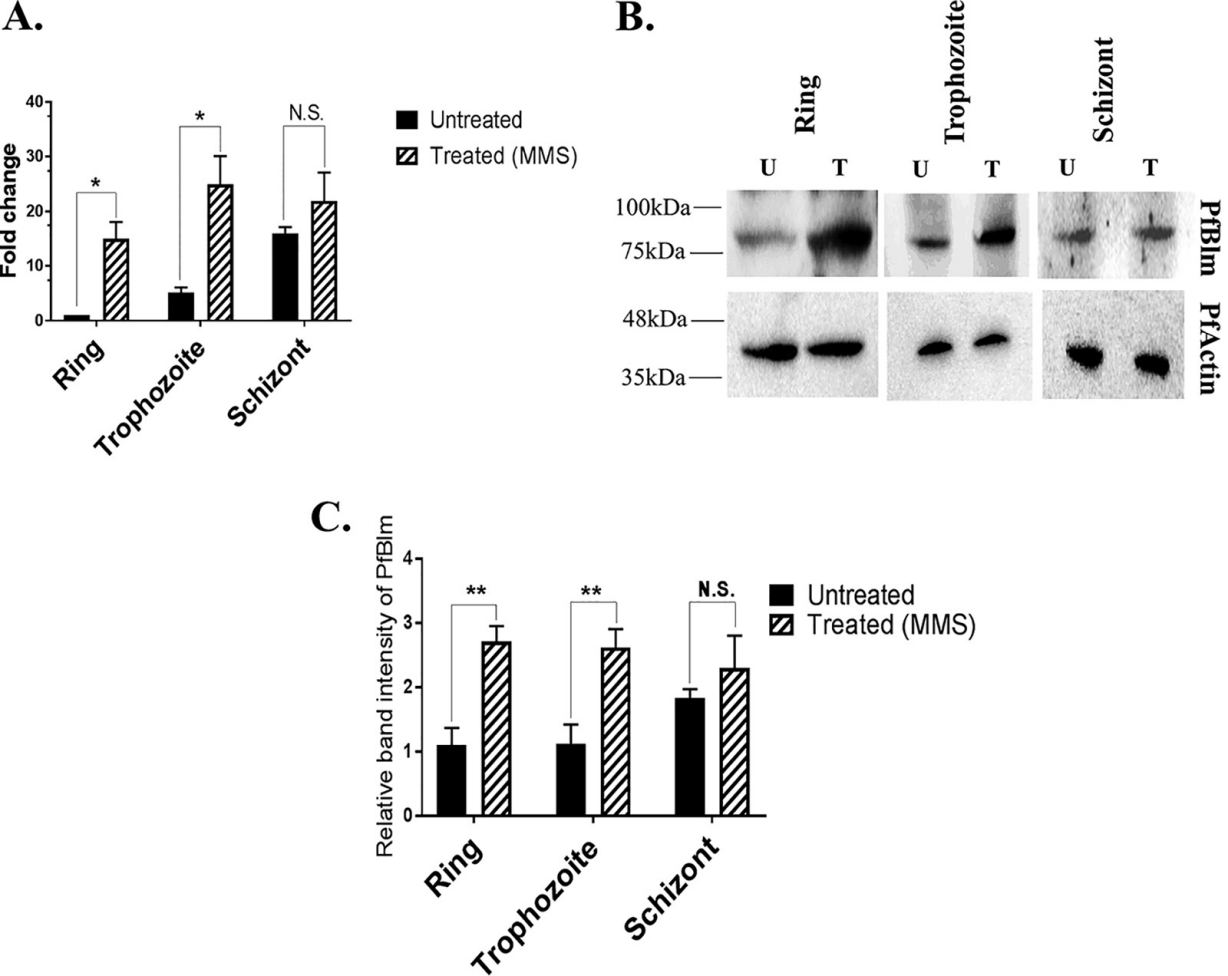
DNA damage-induced upregulation of *PfBLM* during intraerythrocytic developmental stages. (A) Synchronized P. falciparum *in vitro* cultures at the ring, trophozoite, and schizont stages were either untreated (U) or treated (T) with 0.05% MMS for 6 h. Real-time RT-PCR on extracted RNA revealed the upregulation of *PfBLM* mRNA during the ring and trophozoite stages upon DNA damage, but its level was not changed during the schizont stage. *PfARP* transcripts were used as a loading control. Each bar represents the mean value ± SD (*n* = 3). (B) Western blots show that MMS induced the expression of the PfBlm protein at the ring and trophozoite stages, but in the case of the schizont stage, it remained unchanged. PfActin acted as a loading control. The position of molecular markers is indicated on the left. (C) Quantification of Western blot data from three independent experiments. Data were normalized against PfActin. Each bar represents the mean band intensity ± SD (*n* = 3). The *P* value was calculated using the two-tailed *t* test (* means a *P* value of <0.05, ** means a *P* value of <0.01, and N.S. means not significant).

We investigated whether *PfBLM* is overexpressed in response to DNA damage. Previous reports have shown that *Plasmodium* proteins involved in DSB repair are overproduced upon DNA damage (5, 6). Hence, we sought to investigate the stage-specific induction of *PfBLM* under DNA-damaging conditions. To this end, we took tightly synchronous parasites of the ring, trophozoite, and schizont stages and treated them with the known DNA-damaging agent methyl methanesulfonate (MMS) for 6 h. Following this, total RNAs/proteins were isolated from both untreated and treated cultures. We performed real-time RT-PCR to check the expression of *PfBLM* in both treated and untreated cultures. *PfARP* was used as a loading control. We observed a 14-fold upregulation of *PfBLM* under DNA-damaging conditions at the ring stage and a 5-fold upregulation at the trophozoite stage, but its level remained unchanged during the schizont stage (Fig. 2A). We examined the induction of the PfBlm protein by Western blotting in the three blood stages of the parasite. The expression of PfBlm was increased almost 3-fold under DNA-damaging conditions in the ring and the trophozoite stages, whereas no significant induction was observed at the schizont stage (Fig. 2B and C), which correlates with the transcript level. It could be possible that the steady-state level of *PfBLM* mRNA or protein is already so high at the schizont stage that any further induction was not quantifiable. Nonetheless, the upregulation of *PfBLM* in response to DNA damage suggests its likely involvement in DSB repair.

### PfBlm interacts with the key DNA repair proteins PfRad51 and PfalMre11.

**FIG 3:**
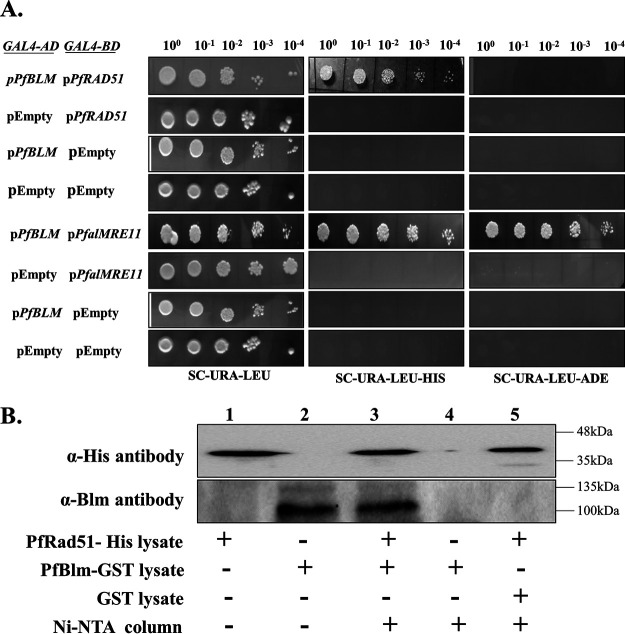
PfBlm interacts with the key DNA repair proteins PfRad51 and PfalMre11. (A) Full-length *PfBLM* was fused to the *GAL4* activation domain (*GAL4-AD*) of pGADC1. Similarly, full-length *PfalMRE11* and *PfRAD51* were fused to the *GAL4* binding domain (*GAL4-BD*) of pGBDUC1. The interaction was tested in yeast strain PJ69-4A, which bears *ADE2* and *HIS* as reporter genes. Starting with the same OD (1 OD/ml), 10-fold serially diluted cells were spotted onto plates lacking leucine and uracil to check for the presence of bait and prey plasmids. Simultaneously, cells were spotted onto plates lacking histidine and adenine to check the interaction. (B) Western blotting shows the interaction between PfRad51 and PfBlm in the Ni-NTA pulldown (lane 3). The cell lysate of histidine-tagged PfRad51 mixed with the empty GST cell lysate (lane 5) did not show a signal with anti-Blm antibody. The description of each lane is shown at the bottom. The position of molecular markers is indicated on the right.

We investigated whether PfBlm physically interacts with other bona fide DSB repair proteins of P. falciparum. In order to function in the DSB repair pathway, PfBlm must interact with proteins of the HR pathway. Previous studies have shown that HsBLM interacts with both HsRad51 and HsMre11 (23, 24). We investigated whether PfBlm interacts with PfRad51 and PfalMre11, which were previously implicated as DNA repair proteins in *Plasmodium* (5, 6). To this end, we performed a yeast two-hybrid analysis. *PfBLM* was cloned into the prey vector harboring the *GAL4* activation domain and the *LEU2* selectable marker. *PfRAD51* or *PfalMRE11* was cloned into the bait vector having the *GAL4* DNA binding domain and *URA3* as a selectable marker. Doubly transformed yeast cells were scored for *HIS3* or *ADE2* reporter gene expression. We observed an interaction between PfBlm and PfRad51 as growth was seen on SC−Leu−Ura−His (synthetic complete medium minus leucine, minus uracil, minus histidine) triple-dropout plates. The interaction with PfalMre11 was found to be much stronger as growth was observed not only on SC−Leu−Ura−His plates but also on SC−Ura−Leu−Ade (synthetic complete medium minus leucine, minus uracil, minus adenine) triple-dropout plates (Fig. 3A). To further confirm the interaction between PfBlm and PfRad51, a copurification assay was performed. To this end, we expressed recombinant PfRad51 with a histidine tag and PfBlm with a glutathione *S*-transferase (GST) tag in Escherichia coli. Both cell lysates were mixed, passed through a Ni-nitrilotriacetic acid (NTA) column, and eluted. We detected both PfBlm and PfRad51 signals in Western blots of the eluates, suggesting their interaction, whereas no signal was detected when only the PfBlm-GST lysate was passed through a Ni-NTA column or the empty GST tag lysate was mixed with histidine-tagged PfRad51 and passed through a Ni-NTA column (Fig. 3B). As full-length *PfalMRE11* could not be expressed in bacterial systems (6), such copurification experiments could not be performed for these pairs of proteins. These observations that PfBlm associates with two well-established DSB repair proteins of P. falciparum strongly suggest a role of PfBlm in the DSB repair pathway in this parasite.

### *PfBLM* functionally complements the MMS sensitivity of an Δ*sgs1* mutant of S. cerevisiae.

**FIG 4:**
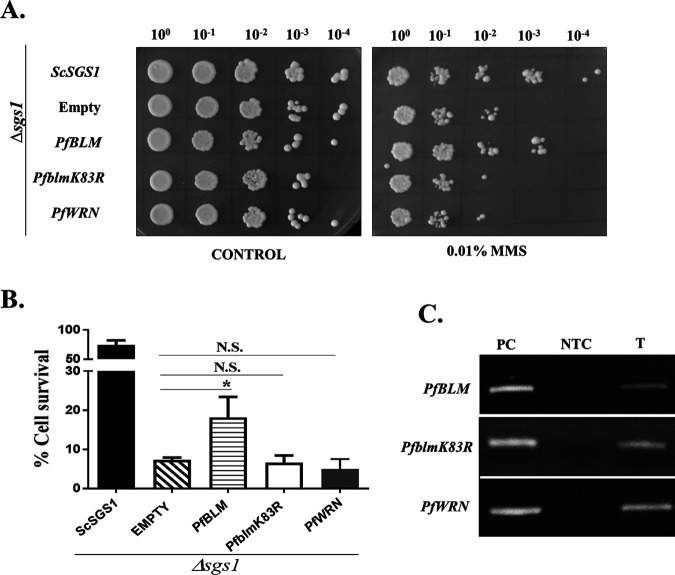
*PfBLM* functionally complements the MMS sensitivity of the Δ*sgs1* mutant of S. cerevisiae. (A) Spotting assay on SC−His plates without (control) or with 0.01% MMS. The genotype of each strain is shown on the left. (B) A return-to-growth assay was performed, where all the complementation strains were either untreated or treated with 0.03% MMS for 2 h and allowed to grow on SC−His plates without MMS after washing. The percentage of cell survival for each strain was obtained by taking the ratio of the numbers of colonies formed between treated and untreated cultures. Each bar represents the mean number ± SD after normalization with untreated controls from three independent experiments (*n* = 3). The *P* value was calculated using the two-tailed *t* test (* means a *P* value of <0.05, and N.S. means not significant). (C) The expression of *PfBLM*, *PfblmK83R*, and *PfWRN* was confirmed by isolating RNA and performing semiquantitative RT-PCR from complementation strains. PC, positive control (*Plasmodium* genomic DNA used as the template); NTC, nontemplate control; T, test (cDNA from the respective complementation strains used as the template).

We investigated whether *PfBLM* can functionally rescue the DNA repair defect of the RecQ mutant of budding yeast. Saccharomyces cerevisiae possesses a single RecQ gene, namely, *SGS1*. The DSB repair properties of Sgs1 are well established. Previous studies have shown that an Δ*sgs1* mutant strain is sensitive to the genotoxic agent MMS (25), and full-length *HsBLM* was able to complement the MMS sensitivity (26). Complementation was dependent on the helicase activity of the HsBLM protein since the helicase-dead mutant *HsblmK695R* was not able to perform the DNA repair function (26). In our study, we investigated whether full-length *PfBLM* and a helicase-dead mutant of *PfBLM* (*PfblmK83R*) can complement the DNA damage sensitivity of the Δ*sgs1* mutant strain. Along with *PfBLM*, another identified RecQ helicase of *Plasmodium*, *PfWRN*, was also tested. To this end, we knocked out the *SGS1* gene from S. cerevisiae to generate the SNY1 strain. *PfBLM*, *PfblmK83R*, and *PfWRN* cloned into the pBFM vector were transformed into SNY1 to generate SNY3, SNY4, and SNY5. We also transformed *ScSGS1* cloned into the pBFM vector and the empty pBFM vector to generate SNY2 and SNY6, which acted as the positive and negative controls, respectively. Complementation studies were done using a return-to-growth assay and by growing strains on plates containing MMS. In both assays, we observed that *PfBLM* could partially complement the DNA damage sensitivity of the Δ*sgs1* mutant, whereas *PfblmK83R* or *PfWRN* was inefficient to overcome the sensitivity (Fig. 4A and B). In the return-to-growth assay, the helicase-dead mutant behaved similarly to the negative control, indicating the importance of the helicase activity of the PfBlm protein in performing the DNA repair function. The expression levels of *PfBLM*, *PfblmK83R*, and *PfWRN* were determined by a semiquantitative RT-PCR method, and the expression levels were found to be comparable to that of *ScSGS1* (Fig. 4C). We used the pBFM plasmid as it would enable us to detect the protein expression of the cloned genes using anti-Myc antibody. However, we failed to detect the expression of any Myc-tagged proteins. These results strongly suggest that the DSB repair function of RecQ helicase is also conserved in PfBlm.

### Overexpression of *PfBLM* provides a survival advantage to the parasites under DNA-damaging conditions.

**FIG 5:**
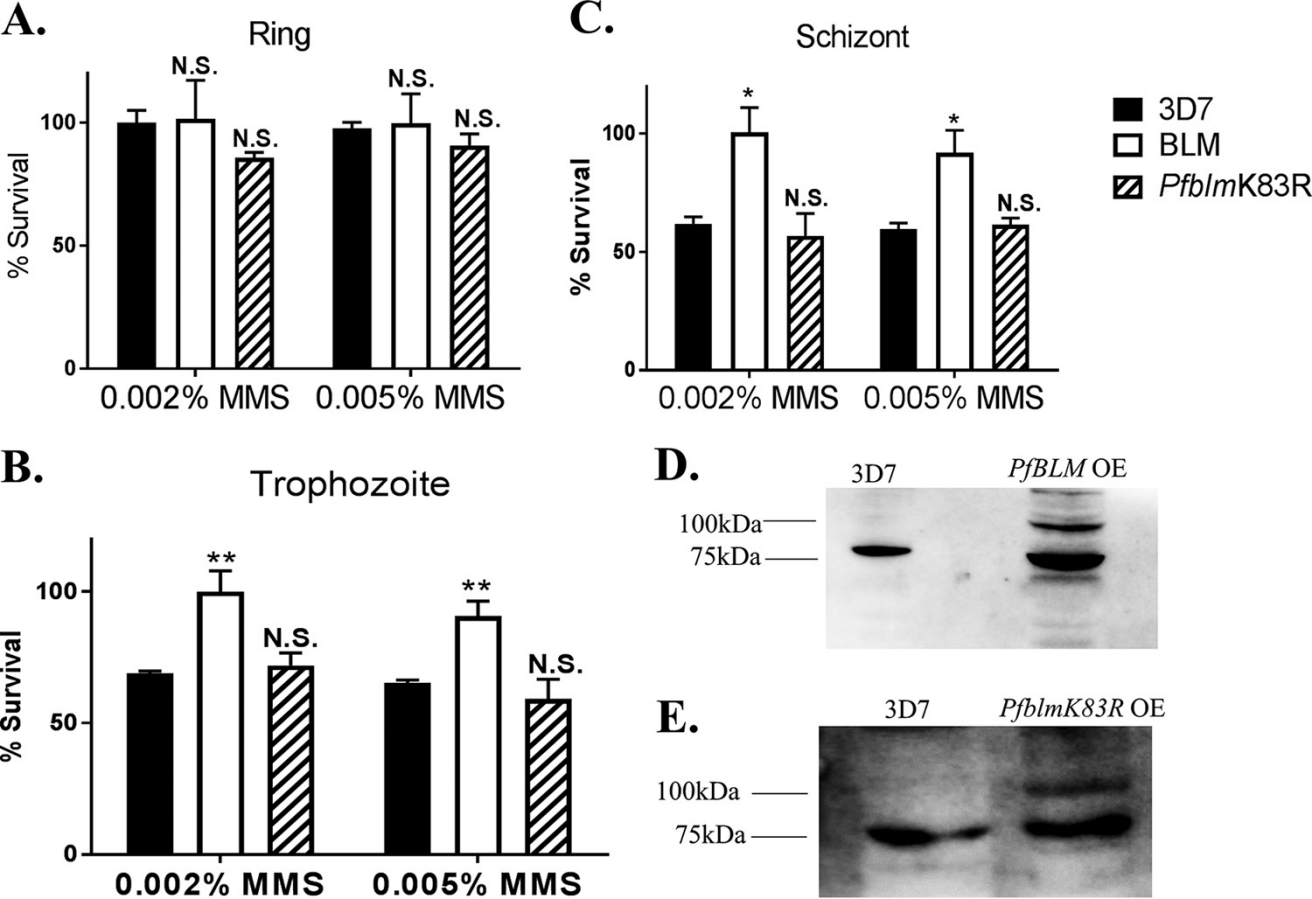
Overexpression of *PfBLM* provides a survival advantage to the parasites under DNA-damaging conditions. (A to C) Plasmodium falciparum 3D7 cells harboring *PfBLM*-GFP and *PfblmK83R-*GFP were either untreated or treated with 0.002% and 0.005% MMS for 2 h and subsequently allowed to grow for 48 h after washing. The experiment was also performed with 3D7 cultures, which acted as a control. After 48 h, smears were prepared, and infected erythrocytes were counted to obtain parasitemia data. The experiment was performed with three intraerythrocytic stages of parasites. Stages are indicated at the top. Percent survival was plotted by taking the ratio of the percentages of parasitemia between the treated and untreated cultures of the respective strains. Each bar represents the mean survival value ± SD (*n* = 3). Significance was calculated with respect to the wild-type strain (3D7). (D) Western blotting shows the expression of the *Pf*Blm protein. The strains are indicated at the top. The expression of the PfBlm (80 kDa) protein in the 3D7 strain and the expression of GFP-tagged PfBlm (107 kDa) protein along with endogenous PfBlm (80 kDa) are shown. OE, overexpression. (E) Expression of the PfBlm (80 kDa) protein in the 3D7 strain (left) and expression of the GFP-tagged PfblmK83R (107 kDa) protein along with endogenous PfBlm (80 kDa) (right). The positions of molecular markers are indicated on the left. The *P* value was calculated using the two-tailed *t* test (* means a *P* value of <0.05, ** means a *P* value of <0.01, and N.S. means not significant).

In order to amplify the function of PfBlm in HR-mediated DSB repair (if any), we performed overexpression studies. The idea behind such experiments was that if PfBlm is involved in the repair of DSBs, the overexpression of *PfBLM* would confer a better repair efficiency than the normal level of the protein. To this end, P. falciparum 3D7 cells were transfected separately with both wild-type *PfBLM* and the *PfblmK83R* mutant cloned into the pARL overexpression vector (27). The transfected parasites at all three intraerythrocytic stages were treated with 0.002% and 0.005% MMS for 2 h and returned to growth for 48 h, after washing off MMS. Simultaneously, the same experiment was performed with a 3D7 culture, which acted as a control. In such experiments, only parasites capable of repairing the DSBs would survive. The survival of the *PfBLM*-overexpressing strain upon DNA damage was found to be significantly better than that of the wild-type 3D7 strain. Interestingly, such survival advantages were observed only for the parasites where DNA damage was induced at the trophozoite or schizont stage but not at the ring stage (Fig. 5A to C). No such survival advantage under DNA-damaging conditions was observed for parasites overexpressing the helicase-dead mutant version of the gene *PfblmK83R* (Fig. 5A to C). The expression of green fluorescent protein (GFP)-tagged PfBlm and PfblmK83R was confirmed by performing Western blotting (Fig. 5D and E). These experiments provide direct evidence for the involvement of PfBlm in the DSB repair pathway of P. falciparum.

### *In silico* analysis of the binding poses and binding affinity of inhibitors against PfBlm.

**FIG 6:**
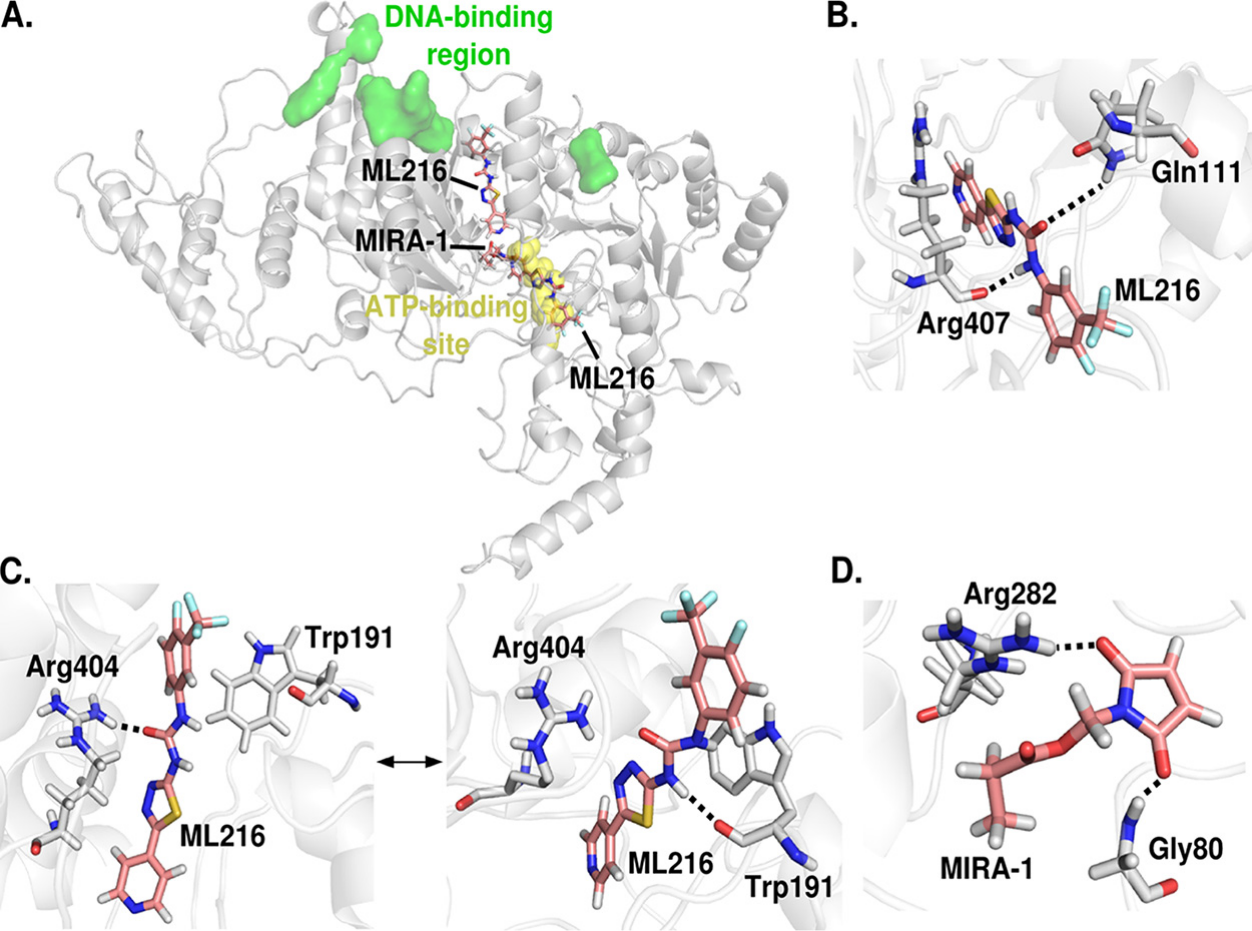
*In silico* analysis of the binding poses and binding affinities of inhibitors against PfBlm. (A) Two binding poses for ML216 and a single binding pose for MIRA-1. ML216 can bind at either the ATP binding site or the DNA binding region, whereas MIRA-1 binds only in the vicinity of the ATP binding site. (B) ML216 interacts with Gln111 and Arg407 in the ATP binding site. (C) ML216 bound near the DNA binding region is seen to exist in two poses: one in which it interacts with Arg404 and one in which it interacts with Trp191. (D) MIRA-1 interacts with the residues Gly80 and Arg282 near the ATP binding site. Hydrogen bonds between the protein and the inhibitor are shown as dotted lines.

We investigated whether chemical inhibitors of RecQ helicases, namely, ML216 and MIRA-1 (28, 29), could potentially bind to PfBlm. To this end, we have taken an *in silico* approach. Since the crystal structure of PfBlm is not available, we have modeled the protein based on the crystal structure of Homo sapiens BLM (HsBlm), the details of which are described in Materials and Methods (see also Fig. S1 in the supplemental material). The inhibitor molecules were first docked onto the PfBlm structure. It was seen that ML216 can bind at either the ATP binding site or the DNA binding region, whereas MIRA-1 can bind only in the vicinity of the ATP binding site (Fig. 6A). In order to evaluate the stability of the docking poses, molecular dynamics simulations of the docked complexes were performed. It was seen that ML216 interacts with two residues, Gln111 and Arg407, in the ATP binding site of PfBlm (Fig. 6B), but when it is bound to the DNA binding region of PfBlm, it is seen to bind with two poses at the same binding site: one in which it interacts with Arg404 and the other in which it interacts with Trp191 (Fig. 6C). MIRA-1 interacts with the residues Gly80 and Arg282 in the vicinity of the ATP binding site (Fig. 6D). An estimate of the binding affinity between PfBlm and the inhibitors was made by calculating the free energy of binding (Table S1). The values show that ML216 has a high affinity for PfBlm, compared to MIRA-1. These results predicted that ML216 could be a potent inhibitor of PfBlm, and hence, a chemical inhibition strategy could be employed to elucidate the function of PfBlm during DSB repair in this parasite.

10.1128/mSphere.00956-20.1 FIG S1 Predicted PfBlm model (shown in gray) superposed with HsBlm (orange). The two structures shown here exhibit high similarity in terms of secondary and tertiary structures. The only region in which they differ corresponds to the missing residues in HsBlm (residues 1194 to 1206) and the adjoining alpha helix (residues 1207 to 1235). Download FIG S1, PDF file, 0.2 MB. Copyright © 2020 Suthram et al. 2020 Suthram et al. This content is distributed under the terms of the Creative Commons Attribution 4.0 International license.

10.1128/mSphere.00956-20.3 TABLE S1 Free energies of binding for inhibitors bound to PfBlm. Download Table S1, PDF file, 0.07 MB. Copyright © 2020 Suthram et al. 2020 Suthram et al. This content is distributed under the terms of the Creative Commons Attribution 4.0 International license.

### Compared to MIRA-1, ML216 is a potent inhibitor of the intraerythrocytic growth of P. falciparum.

**FIG 7:**
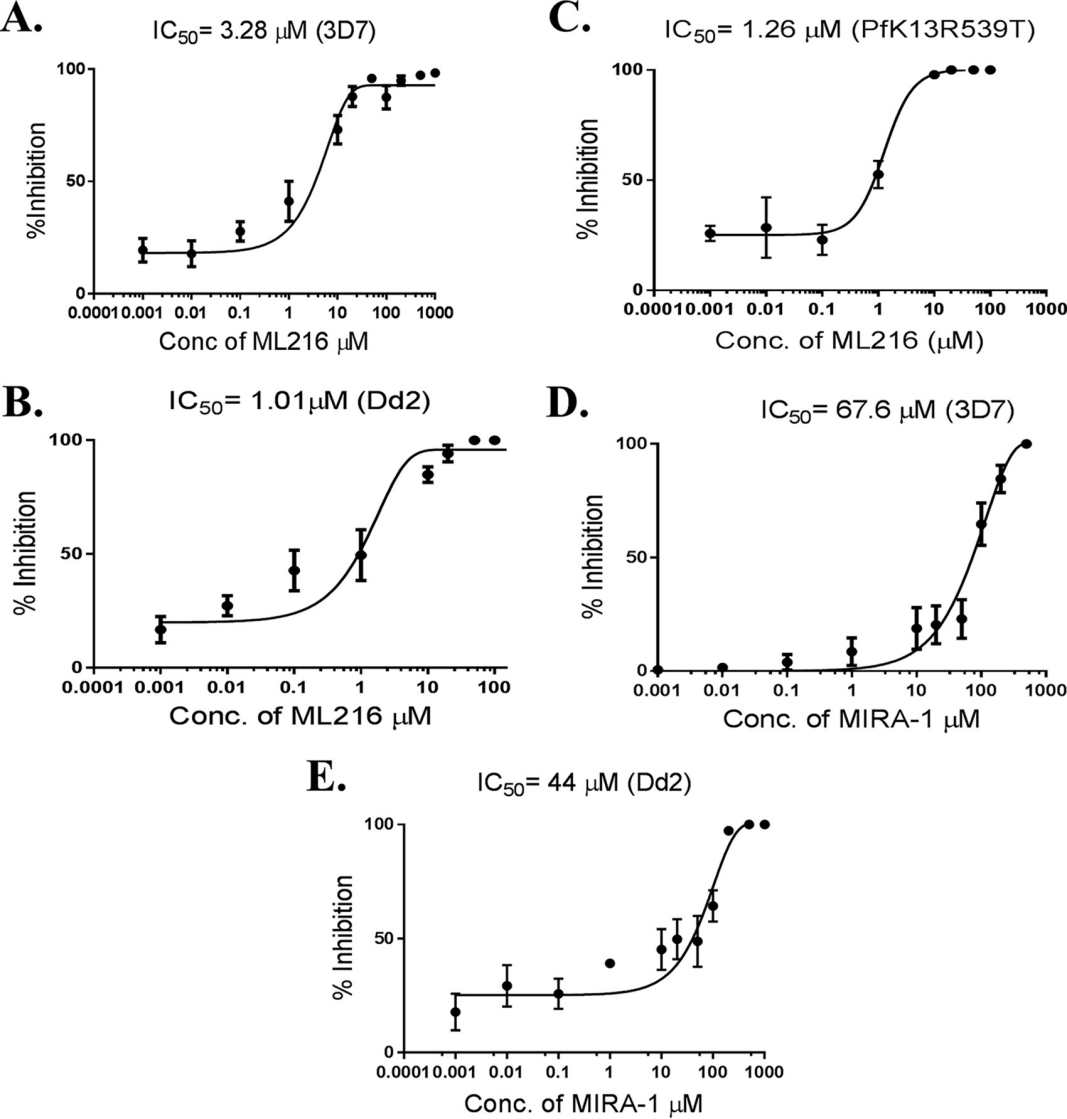
Compared to MIRA-1, ML216 is a potent inhibitor of the intraerythrocytic growth of P. falciparum. We employed a Giemsa staining method to evaluate percent parasitemia and deduced percent inhibition from the obtained parasitemia values. (A) Synchronous trophozoite-stage parasites (3D7) were grown for 48 h in the presence of various concentrations of ML216. Parasite growth inhibition at various concentrations of the drug was plotted to obtain the IC_50_ values. (B) Growth inhibition with ML216 in Dd2 parasites was plotted to obtain IC_50_ values. (C) Growth inhibition with ML216 in PfK13R539T parasites was plotted to obtain IC_50_ values. (D) The effect of MIRA-1 on 3D7 parasites was tested according to the same protocol as the one for ML216 treatment. Percent inhibition was plotted against various concentrations of the drug to obtain the IC_50_ values. (E) Growth inhibition with MIRA-1 in Dd2 parasites was plotted to obtain the IC_50_ values. Each assay was repeated three times for reproducibility (*n* = 3).

To study the effect of RecQ helicase inhibitors (ML216 and MIRA-1) on *Plasmodium* growth, we performed a growth inhibition assay in the presence of different concentrations of both drugs. RecQ helicases are involved in replication and DSB repair in a variety of organisms. Malaria parasites undergo several rounds of replication during erythrocytic schizogony, and repair of endogenous DNA damage is a common occurrence in the life cycle of *Plasmodium*. We investigated whether any of the above-mentioned RecQ inhibitors actually inhibit the blood-stage development of the parasite. We treated trophozoite cultures with ML216 or MIRA-1 at various concentrations (1 nM to 1,000 μM) for 48 h and assessed parasitemia by staining the smears with Giemsa stain. Both ML216 and MIRA-1 were able to inhibit the intraerythrocytic developmental cycle (IDC) of the parasites. However, ML216 was more potent than MIRA-1. The dose-response curve yielded 50% inhibitory concentration (IC_50_) values of 3.28 μM and 67.6 μM for ML216 and MIRA-1 (Giemsa staining), respectively (Fig. 7A and D). We also employed a fluorescence-based SYBR green I method to estimate survival. This method yielded IC_50_ values of 6.31 μM for ML216 and 55.2 μM for MIRA-1 (Fig. S2A and D). We further tested the effects of ML216 and MIRA-1 on the drug-resistant strain Dd2. We observed that the IC_50_ values are in similar ranges albeit lower than what was observed for the drug-sensitive 3D7 strain. For ML216, the observed IC_50_ value was 1.01 μM, and for MIRA-1, the value was 44 μM (Giemsa staining) (Fig. 7B and E). The SYBR green I method yielded IC_50_ values of 3.24 μM for ML216 and 58 μM for MIRA-I (Fig. S2B and E). In addition, we also examined the inhibitory effect of ML216 on the artemisinin-resistant strain PfK13R539T (30). The Giemsa staining method yielded an IC_50_ value of 1.26 μM (Fig. 7C), and with the SYBR green I method, the IC_50_ value was 2.19 μM (Fig. S2C). The IC_50_ values obtained by both the Giemsa staining method and the SYBR green I method are in similar ranges. Thus, ML216 was found to be a more potent inhibitor of the IDC than MIRA-1.

10.1128/mSphere.00956-20.2 FIG S2 Compared to MIRA-1, ML216 is a potent inhibitor of the intraerythrocytic growth of P. falciparum. The SYBR green I method was employed to measure the growth of parasites. (A) Parasite growth inhibition with various concentrations of ML216 in 3D7 was plotted to obtain IC_50_ values. The inset depicts the standard curve of parasitemia versus the SYBR green I fluorescence intensity. (B and C) Growth inhibition with ML216 in Dd2 and PfK13R539T parasites was plotted to obtain IC_50_ values. (D and E) Dose-response curves plotted to obtain IC_50_ values of MIRA-1 in the 3D7 and Dd2 strains by the SYBR green I method. Download FIG S2, PDF file, 0.06 MB. Copyright © 2020 Suthram et al. 2020 Suthram et al. This content is distributed under the terms of the Creative Commons Attribution 4.0 International license.

### Parasites treated with ML216 become hypersensitive to the DNA-damaging agent MMS.

To investigate the effect of ML216 on DNA-damaged cells, we performed a growth inhibition assay with MMS-treated cells. Previous studies have shown that MMS creates numerous double-strand breaks and that such breaks are repaired over the period (31). However, if such breaks are not successfully repaired, cells bearing the unrepaired breaks succumb to death. Thus, the MMS sensitivity assay is a good measure of the DSB repair capability of the cells. We reasoned that inhibition of PfBlm with ML216 would render parasites more sensitive to MMS treatment. To this end, we treated synchronized parasite cells of all stages with various concentrations of ML216 (1 nM to 1,000 μM) in the presence or absence of MMS. The assay was performed in both drug-sensitive (3D7) and drug-resistant (Dd2) strains. MMS treatment drastically lowered the IC_50_ value of ML216 for both the 3D7 and Dd2 parasite strains (Table 1). The effect was most prominent when DSBs were induced at the trophozoite stage. The drops in the IC_50_ values at the trophozoite stage were 264-fold and 218-fold for 3D7 and Dd2, respectively. The creation of DSBs at the schizont stage also had a profound effect on the IC_50_ value of ML216. There were about 185-fold and 40-fold reductions in the IC_50_ values for the 3D7 and Dd2 strains, respectively. The effect on the ring stage was very minimal, with only a 2- to 2.5-fold reduction. Such dramatic drops in the IC_50_ values are not due to an additive effect of MMS toxicity as the survival rates of the ring-, trophozoite-, and schizont-stage parasites against MMS are 97%, 65%, and 60%, respectively (Fig. 5A to C). These results imply that in the presence of external DNA-damaging agents, ML216 could be an effective inhibitor of parasite growth as it works at the nanomolar range.

**TABLE 1:** IC_50_ values of ML216 with or without MMS in treated cultures at different IDC stages in the 3D7 and Dd2 strains

Stage and strain	Treatment	IC_50_ (μM)
Ring		
3D7	ML216 (alone)	3.49
	ML216 + MMS	1.42
Dd2	ML216 (alone)	1.85
	ML216 + MMS	0.96

Trophozoite		
3D7	ML216 (alone)	2.8
	ML216 + MMS	0.0106
Dd2	ML216 (alone)	0.871
	ML216 + MMS	0.004

Schizont		
3D7	ML216 (alone)	3.21
	ML216 + MMS	0.0174
Dd2	ML216 (alone)	1.49
	ML216 + MMS	0.0374

### ML216 blocks the repair of UV-induced DNA damage in the nuclear genome of the parasite.

**FIG 8:**
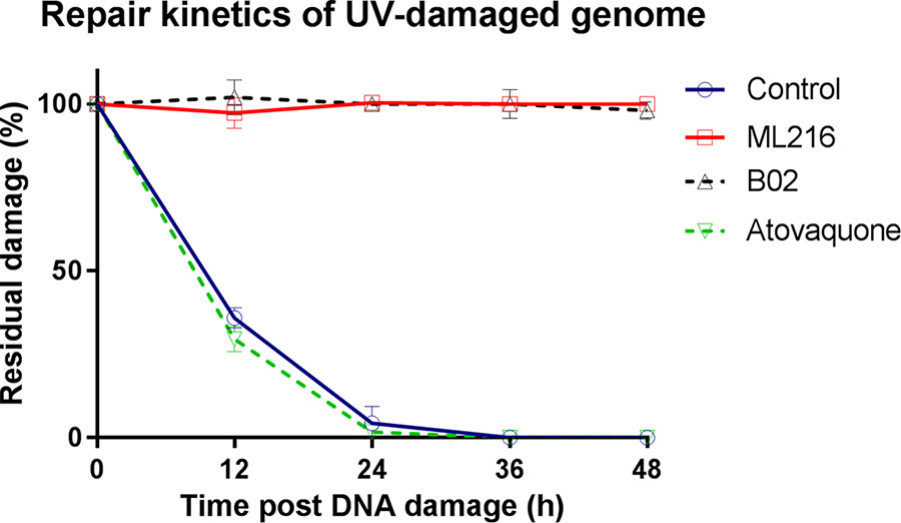
ML216 blocks the repair of UV-induced DNA damage in the nuclear genome of the parasite. ML216-, B02-, and atovaquone-pretreated and mock-treated cultures were UV irradiated at 100 J/m^2^ at the trophozoite stage. After UV treatment, cells were grown in the presence of sublethal doses of the respective drugs (ML216, B02, or atovaquone) for 48 h. The mock-treated culture was also grown in parallel in normal complete medium, which acted as the control. Genomic DNA was isolated before and after treatment (untreated and 0 h posttreatment) and at 12-h intervals until 48 h. The percentage of unrepaired damage was plotted at the indicated time points using GraphPad Prism software.

Next, we investigated the effect of ML216 on the repair of DNA breaks in the parasite genome. To this end, we employed a PCR-based technique to estimate the amount of UV-induced damage at a particular region of the parasite genome as defined by the position of PCR primers (32). This method relies on the fact that a longer stretch of genomic DNA is more likely to experience UV-induced damage, and for a very short stretch of the genome, such a probability is negligible. Thus, a decrease in the long-range PCR product would indicate the accumulation of DNA damage in the template DNA. As the amount of the short-range PCR product is expected to remain unchanged before and after DNA damage, this can be used to normalize the data. We treated the cultures with a sublethal concentration of ML216, monitored the repair kinetic of UV-induced DNA damage, and compared it with the repair kinetic of the untreated parasites. As a positive control, we used B02, a known small-molecule inhibitor of the *Plasmodium* DSB repair pathway. B02 is a potent inhibitor of PfRad51, and hence, it blocks the repair of DSBs in the *Plasmodium* genome (33). As a negative control, we included atovaquone (ATQ) in our study, as this chemical is not related to DSB damage or repair. We observed that in the untreated parasites, DNA damage is repaired by 24 h, but in the parasites treated with ML216, the damage remained unrepaired even after 48 h (Fig. 8). Similar kinetics were observed with parasites treated with B02. Atovaquone treatment did not have any inhibitory effect on the repair of UV-induced DNA damage. Thus, these results strongly suggest that the Blm inhibitor ML216 is also a potent inhibitor of *Plasmodium* DSB repair.

### ML216 interacts synergistically with ART and CQ.

**FIG 9:**
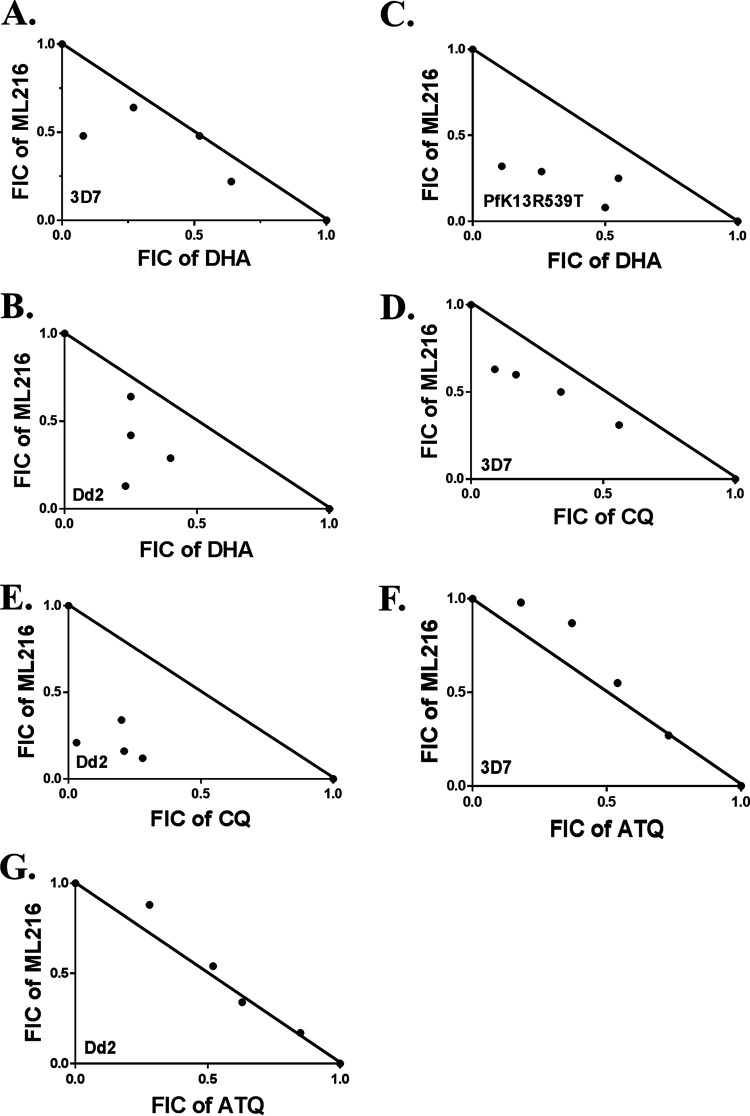
ML216 interacts synergistically with ART and CQ. (A to C) Isobolograms of dihydroartemisinin (DHA)-ML216 in the 3D7 (A), Dd2 (B), and PfK13R539T (C) strains. (D and E) Isobolograms of chloroquine (CQ)-ML216 in the 3D7 (D) and Dd2 (E) strains. (F and G) Isobolograms of atovaquone (ATQ)-ML216 in the 3D7 (F) and Dd2 (G) strains. Fixed-ratio drug combination assay. Each point represents the mean IC_50_ of the drug combination from three independent experiments. A solid line is plotted between the IC_50_ values of each drug when used alone. FIC, fractional inhibitory concentration.

We studied the interaction of ML216 with the known antimalarial drug ART. Since ART creates DSBs (34), and in the presence of ML216, the repair of such breaks is inhibited, we hypothesized that ML216 and ART might potentiate each other’s action. To that end, we performed a fixed-ratio drug combination assay. Dose-response curves were plotted for each combination, and fractional inhibitory concentration (FIC) values were calculated from the graphs. The ΣFIC values are tabulated in Table S2. Finally, an isobologram was constructed based on the calculated ΣFIC values. As depicted in the isobologram, the interaction between ML216 and ART is synergistic in nature (Fig. 9A). A similar result of a synergistic relationship was observed for the drug-resistant strains Dd2 and PfK13R539T (Fig. 9B and C). As chloroquine (CQ) treatment inhibits heme polymerization leading to the generation of free radicals, it is speculated that the treatment of parasites with CQ is also likely to create DSBs in the parasite (35). As expected, we observed that ML216 also synergizes with CQ in both drug-sensitive and drug-resistant strains (Fig. 9D and E). We also tested the interaction of ML216 with ATQ, whose mode of action does not interfere with DNA metabolism. As expected, we did not observe any synergistic action between these two drugs (Fig. 9F and G). These results suggest that the action of both ART and CQ can be potentiated by the RecQ inhibitor ML216.

10.1128/mSphere.00956-20.4 TABLE S2 FIC values of drug combinations. Download Table S2, PDF file, 0.08 MB. Copyright © 2020 Suthram et al. 2020 Suthram et al. This content is distributed under the terms of the Creative Commons Attribution 4.0 International license.

## DISCUSSION

In this study, we established one of the RecQ helicases of *Plasmodium*, PfBlm, as a DNA repair protein, and we have shown that a RecQ helicase inhibitor blocks the repair of ART-generated DSBs in the *Plasmodium* genome. First, the expression level of *PfBLM* was high during the schizont stage and upregulated in response to DNA damage. Second, this protein is able to complement the MMS sensitivity of an Δ*sgs1* mutant of S. cerevisiae and interacts with DNA repair proteins of *Plasmodium*. Third, the overexpression of this protein increased the survival of parasites upon DNA damage. Finally, a chemical inhibitor of this protein, ML216, impaired the growth of parasites, and the inhibitor interacted synergistically with first-line antimalarial drugs.

The expression of PfBlm was at its peak during the replicative stage (schizont) compared to the trophozoite and ring stages. A possible interpretation of this result is that RecQ helicases play a crucial role in rescuing stalled replication forks (36), and in a recent study of *Plasmodium*, it has been shown that replication forks stalled at elevated rates in a Δ*Pfblm* strain (18), indicating the active role of PfBlm in replication and repair. The upregulation of this protein in the presence of an external DNA-damaging agent implies its role in DNA repair, and it is in congruence with previous reports in which the DNA repair proteins PfRad51 and PfalMre11 were also upregulated (5, 6). We observed that PfBlm associates with PfRad51 and PfalMre11. The interaction with PfRad51 as scored by yeast two-hybrid assays was found to be weak. It could be possible that within the parasites, certain posttranslational modifications may modulate the strength of such an interaction, which is absent in yeast. It is known from studies in other organisms that depending upon the posttranslational modification of HsBLM, the consequence of its interaction with Rad51 can be either pro- or antirecombinogenic (11). Previous reports suggest that Mre11 helps in the recruitment of HsBLM to DNA ends, thereby stimulating BLM-DNA2-RPA-mediated resection of DSBs (9). PfRPA has been characterized (31), and from the genome information, the putative ortholog of DNA2 has been annotated in P. falciparum (gene identifier PF3D7_1106700) (37). Future experiments in *Plasmodium* may enlighten us about the importance of the PfBLM-PfDNA2-PfRPA interaction.

In the current study, using a yeast surrogate system, we have shown that *PfBLM* rescued the MMS sensitivity phenotype of the Δ*sgs1* strain. However, the helicase-dead mutants of *PfBLM* (*PfblmK83R*) and *PfWRN* were unable to perform this function. In model organisms, the helicase activity of BLM is required for two steps of HR: one during the resectioning of DSB ends through the DNA2-mediated pathway and the other during the dissolution of Holliday junctions along with TopoIII. Although the orthologs of all these genes are annotated in the *Plasmodium* genome, whether PfBlm can engage with PfDNA2 or PfTopoIII and participates at these two distinct steps of HR needs to be experimentally tested. Nonetheless, our results indicate that the helicase activity of *PfBLM* is important in repairing damaged DNA. Previous reports have shown that human *WRN* failed to rescue the MMS sensitivity of the Δ*sgs1* strain, indicating that its function is dispensable for DSB repair (38). This holds true even for *PfWRN*, which was also unable to complement the MMS sensitivity phenotype of the Δ*sgs1* strain. In model organisms, it was found that the RecQ helicases possess both prorecombination and antirecombination functions (7, 8). A previous study observed increased recombination frequencies among the subtelomeric *var* genes in a *Pfwrn* knockdown parasite line but not in a *Pfblm* knockout parasite line (18), emphasizing the antirecombination activity of the PfWrn protein. Our study provides evidence of the prorecombination activity of the PfBlm protein. Thus, it is possible that the two RecQ paralogs of *Plasmodium* may have opposing roles in HR. It is also possible that as observed in model organisms, the pro- and antirecombination functions of these two proteins may be regulated via posttranslational modifications (11, 39–42). Such possibilities need to be explored in the future.

We observed that the overexpression of *PfBLM* reduced the MMS sensitivity of parasites. This finding indicates the DNA repair activity of *PfBLM* in the parasite. A plausible explanation for the increase in the repair efficiency of PfBlm might be its involvement in the unwinding of different substrates of the HR pathway as the overexpression of the helicase-dead mutant did not provide any advantage. BLM also possesses a helicase-independent stimulatory function in the Exo1-mediated resection step during HR. Although *PfEXO1* has been annotated (PF3D7_0725000), we currently do not know whether the overexpression of PfBlm also stimulates such a pathway in *Plasmodium*.

Here, we report the binding affinities of the known RecQ helicase inhibitors ML216 and MIRA-1 for PfBlm by performing docking and molecular dynamics simulations. Our results demonstrated that ML216 binds efficiently to PfBlm compared with MIRA-1. This result is in accordance with the IDC inhibition of parasites with both drugs. The parasites are more sensitive to ML216 than to MIRA-1. Interestingly, ML216-mediated inhibition seems to be more selective toward *Plasmodium* than human fibroblast cell lines, where the half-maximal inhibitory concentration was more than 50 μM after 48 h of incubation (28). Given the strong selectivity of ML216 toward *Plasmodium*, we believe that chemical modification of the lead compound may bring antiparasitic activity in the nanomolar range. Notably, multidrug-resistant strain Dd2 was more sensitive to ML216, and a similar effect was observed with the PfRad51 inhibitor B02 (33). The likely explanation for this result could be that the Dd2 strain might have a compromised DNA repair system, and recent reports have shown that the Dd2 strain failed to respond to DNA damage induced by MMS (43).

Failure in the repair of DSBs inflicted by various endogenous sources such as metabolites and reactive oxygen species (ROS) and replication errors during schizogony lead to the death of parasites. The possible mechanism for the antiparasitic activity of ML216 is to hinder the repair of such endogenous damage. Consistent with this interpretation, the effect of the drug should intensify in the presence of external DNA damage. Our growth inhibition experiment with MMS-treated cells confirmed this hypothesis, with the parasite being hypersensitive to lower concentrations of the drug. The known antimalarial drug ART inhibits different cellular pathways of parasites, and creating DSBs is one such established mode of action among them. Our laboratory has taken a particular interest in blocking the repair of such ART-generated DSBs (34) with ML216. Indeed, ML216 was able to inhibit the repair of DSBs, offering a ray of hope for artemisinin-based combination therapy (ACT). Finally, our results demonstrated that ML216 interacts synergistically with the first-line antimalarial drugs ART and CQ. The plausible explanation for the CQ-ML216 synergistic action might be that CQ inhibits heme polymerization resulting in the generation of free radicals, which potentially creates DSBs. However, there is no experimental evidence for this hypothesis.

Altogether, our data provide compelling evidence for PfBlm’s participation in *Plasmodium* DNA repair. In addition, we have shown that RecQ helicases in *Plasmodium* can be targeted given their functions in different DNA metabolic pathways. This study raises the possibility of using RecQ helicase inhibitors in combination with the first-line antimalarial drugs ART and CQ.

## MATERIALS AND METHODS

Dihydroartemisinin (DHA) was used as an artemisinin (ART) derivative. DHA, CQ, ML216, and MIRA-1 were purchased from Sigma-Aldrich. DHA, ML216, and MIRA-1 were dissolved in dimethyl sulfoxide (DMSO). CQ was dissolved in MilliQ water.

### *Plasmodium* culture.

The 3D7 or Dd2 strain of P. falciparum was grown in human erythrocytes (RBCs) in RPMI 1640 medium supplemented with 0.005% hypoxanthine and 1% Albumax. The hematocrit of RBCs was maintained at 5%.

### Plasmids.

For complementation assays, *PfBLM*, *PfblmK83R*, and *PfWRN* were cloned into the yeast expression vector pBFM. pBFM is a 2μ yeast expression vector prepared by fusing the promoter region of the pTA vector (44) and the vector backbone from the pESC-HIS plasmid (Agilent Technologies). P. falciparum 3D7 genomic DNA was used as the template for the amplification of *Plasmodium* genes. The *PfblmK83R* mutant was generated by splicing by overlap extension PCR. A list of primers used in this study is given in Table S3 in the supplemental material.

10.1128/mSphere.00956-20.5 TABLE S3 Primers used in this study. Download Table S3, PDF file, 0.03 MB. Copyright © 2020 Suthram et al. 2020 Suthram et al. This content is distributed under the terms of the Creative Commons Attribution 4.0 International license.

For yeast two-hybrid analyses, pGBDU-C1 was used as the bait vector, and pGAD-C1 was used as the prey vector, which are 2μ plasmids having *URA3* and *LEU2* markers, respectively (3). *PfRAD51* and *PfalMRE11* were cloned into the bait vector containing the N-terminal *GAL4* DNA binding domain, generating the *PfRAD51*-BD and *PfalMRE11*-BD fusions, respectively. *PfBLM* and *PfWRN* were cloned into the prey vector containing the N-terminal *GAL4* activation domain, generating the *PfBLM*-AD and *PfWRN*-AD fusions, respectively.

For copurification assays, *PfBLM* was cloned into the pGEX-6P2 vector (GE Healthcare Life Sciences), and *PfRAD51* was cloned into the pET101D vector (45).

For overexpression studies in *Plasmodium*, *PfBLM* and *PfblmK83R* were cloned into the pARL-GFP vector (27).

### Yeast strains.

Vector pBFM harboring *ScSGS1*, *PfBLM*, *PfWRN*, or *PfblmK83R* was transformed into the Δ*sgs1* strain SNY1 to generate the SNY2, SNY3, SNY4, and SNY5 strains. The empty pBFM vector was transformed into SNY1 to generate SNY6. Yeast two-hybrid analysis was performed using the PJ69-4A strain. Bait and prey plasmids were transformed into this strain to check the interaction of proteins. A list of yeast strains with genotypes is given in Table S4.

10.1128/mSphere.00956-20.6 TABLE S4 Yeast strains used in this study. Download Table S4, PDF file, 0.09 MB. Copyright © 2020 Suthram et al. 2020 Suthram et al. This content is distributed under the terms of the Creative Commons Attribution 4.0 International license.

### RNA isolation and RT-PCR.

Synchronous P. falciparum cultures were harvested at the ring, trophozoite, or schizont stage when parasitemia was between 8 and 10%. For stage-specific DNA damage experiments, cells were harvested from untreated and MMS-treated cultures. RNA isolation was done as mentioned previously (6). Similarly, total RNA was isolated from yeast strains SNY3 to SNY5 after incubation at 30°C using an acid-phenol method as described previously (44). Equal amounts of RNA were taken after estimating the concentration by spectroscopic analysis (EMC-709 spectrophotometer; JASCO) and subjected to DNase I (Fermentas) treatment to eliminate genomic DNA contamination. PCR was performed to confirm the absence of genomic DNA. The synthesis of cDNA was performed as described previously (44). Briefly, 10 μg of total RNA was reverse transcribed using reverse transcriptase (Qiagen), and the cDNA product was then subjected to real-time RT-PCR. cDNA was diluted 1:10 and subjected to real-time PCR using SYBR Premix ExTaq (TaKaRa) and the Applied Biosystems 7500 Fast real-time PCR system. The threshold cycle (*C_T_*) value of *PfARP* for each sample was normalized to the corresponding *C_T_* value of *PfBLM* transcripts. After normalization, *C_T_* values of *PfBLM* for different samples were compared to obtain Δ*C_T_* values. The 2^Δ^*^CT^* formula was used to determine the relative levels of *PfBLM* mRNA. Each experiment was repeated three times (*n* = 3), and the *P* value was calculated using the two-tailed *t* test (* means a *P* value of <0.05, ** means a *P* value of <0.01, *** means a *P* value of <0.001, and N.S. means not significant).

### Antibodies and Western blotting.

The anti-PfBlm antibody was generated by selecting a unique peptide at the C terminus of the PfBlm protein ( KELEKREEELNEKTKNDQE), and antibody was raised against the peptide in rabbit. Anti-PfBlm antibody, anti-hAct1 antibody (Abcam), and anti-His tag antibody (Invitrogen) were used at a 1:5,000 dilution. Horseradish peroxidase (HRP)-conjugated anti-rabbit secondary antibody (Calbiochem) was used at a 1:10,000 dilution. HRP-conjugated anti-mouse secondary antibody (Calbiochem) was used at a 1:10,000 dilution for actin and His-tagged proteins. Western blotting was performed as previously described (3). The Western blots were developed using a chemiluminescence system (Thermo Scientific), and the band intensities were quantified using ImageJ software. Each experiment was repeated three times (*n* = 3), and the *P* value was calculated using the two-tailed *t* test (* means a *P* value of <0.05, ** means a *P* value of <0.01, *** means a *P* value of <0.001, and N.S. means not significant).

### DNA damage experiments in yeast.

Spotting assays with yeast strains SNY2 to SN6 were performed according to a previously mentioned protocol (6). Briefly, complementation strains were grown in medium lacking histidine at 30°C to an optical density at 600 nm (OD_600_) of 0.5. After normalization and serial dilution of the cells, spotting was done on an SC−His plate containing 0.01% MMS and an SC−His plate without MMS, which acted as the untreated control. The plates were incubated at 30°C, and their growth was compared. A return-to-growth assay with yeast strains SNY2 to SNY6 was performed as previously described (3). Briefly, yeast strains SNY2 to SNY6 were grown until the logarithmic phase and then divided into two equal parts. One part was exposed to 0.03% MMS for 2 h, whereas the other part was incubated without any treatment. The MMS was then washed off, and equal numbers of cells from both parts for each strain were spread on an SC−His plate and incubated for 48 h at 30°C. Percent survival was determined by taking the ratio of the numbers of colonies formed in treated versus untreated cultures. Each experiment was repeated three times (*n* = 3), and a graph was plotted using GraphPad Prism software. The *P* value was calculated using the two-tailed *t* test (* means a *P* value of <0.05, ** means a *P* value of <0.01, *** means a *P* value of <0.001, and N.S. means not significant).

### Yeast two-hybrid analyses.

A yeast two-hybrid analysis was performed as described previously (3). Briefly, the interaction between bait and prey fusion constructs was analyzed by assessing the growth of the SNY8, SNY11, SNY9, SNY12, SNY13, and SNY15 strains on SC−Ura−Leu−His and SC−Ura−Leu−Ade triple-dropout plates. The plates were incubated at 30°C for 5 days.

### Copurification assay.

For performing copurification assays, Rosetta(DE3) cells harboring pGEX6P2:*PfBLM* were induced with 1 mM isopropyl-thiogalactosidase (IPTG) at 16°C overnight, whereas the same cells harboring pET101D:*PfRAD51* were induced with 1 mM IPTG at 37°C for 4 h. Lysis and Ni-NTA purification were performed as described previously (46). Similarly, extracts of cells expressing recombinant PfRad51 and an empty GST tag were also processed in the same manner, which acted as controls. The eluted samples were further analyzed by Western blot techniques.

### Transfection in P. falciparum.

Tightly synchronized ring-stage *Plasmodium* parasites having a parasitemia of around 6% were transfected with 100 μg of the *PfBLM*-p*ARL* and *PfblmK83R*-p*ARL* constructs according to a protocol described previously (47). Briefly, 1 day before transfection, both plasmids were resuspended in 50 μl of a Cytomix solution (10 mM K_2_HPO_4_ [pH 7.6], 120 mM KCl, 0.15 mM CaCl_2_, 25 mM HEPES [pH 7.6], 2 mM EGTA [pH 7.6], and 5 mM MgCl_2_) and kept at 4°C overnight. Electroporation was done using a Bio-Rad gene pulser. After transfection, cells were allowed to grow in the absence of the drug until parasitemia reached 4%. Later, cells were maintained in drug medium until transfectants appeared. Pyrimethamine was used as a selectable drug.

### DNA damage experiments in parasites.

DNA damage-specific expression of *PfBLM* was done by treating cells with 0.05% MMS for 6 h, and untreated cells were grown simultaneously. RNA/protein was isolated from both groups of cells for real-time RT-PCR and Western blotting.

For survival assays with the overexpression of *PfBLM* and *PfblmK83R*, parasites were synchronized, and parasites having a parasitemia of around 1% were taken for treatment with 0.002% or 0.005% MMS for 2 h. After 2 h of treatment with MMS, cells were washed twice and allowed to grow for 48 h in complete medium. Wild-type parasites (3D7) were also given MMS treatment in a similar manner, which served as controls. The ratio of the percentages of survival between the transfectant and the wild type was taken to plot the fold survival advantage. Each experiment was repeated three times, and the graph was plotted using GraphPad Prism software. The *P* value was calculated using the two-tailed *t* test (* means a *P* value of <0.05, ** means a *P* value of <0.01, *** means a *P* value of <0.001, and N.S. means not significant).

### Docking and molecular dynamics simulations.

The PfBlm protein was modeled using the I-TASSER server (48, 49). A quality check of the predicted structural models was performed using the PROCHECK program (50). The model closest to the HsBLM structure (PDB accession number 4CGZ) (51) in terms of the root mean square deviation (RMSD) was chosen for further studies. A 10-ns equilibration of the model was carried out using the GROMACS program with the CHARMM36 force field (52, 53). In order to choose a representative structure from the simulation trajectory, clustering of structures from the last 5 ns of the trajectory was performed using the GROMACS program. The structure at the center of the largest cluster was chosen as a representative structure for docking studies. The inhibitors ML216 and MIRA-1 were docked onto the PfBlm model using the AutoDock Vina program (54). Two independent docking runs were performed for both inhibitors: one with the ATP binding site as the docking search space and one with the DNA binding region as the docking search space. For each run, the docking pose with the highest number of protein-inhibitor hydrogen bonds was selected for molecular dynamics simulations. The protein-inhibitor complex was simulated for 50 ns using the GROMACS program with the CHARMM36 force field (52, 53). Force field parameters for the inhibitor molecules were obtained using the Paramchem server (55, 56). The binding free energy for the protein-inhibitor complex was calculated using the molecular mechanics Poisson-Boltzmann surface area (MM-PBSA) method (57, 58).

### Inhibition of *Plasmodium* growth by ML216 and MIRA-1.

Drug inhibition assays with ML216 and MIRA-1 were performed as previously described (33). Both Giemsa staining and SYBR green I-based evaluation of parasite survival were done as previously described (59). The semilog graph was plotted for the concentration of the drug versus the percent inhibition to determine the 50% inhibitory concentrations (IC_50_s) of ML216 and MIRA-1 using GraphPad Prism software. Each assay was repeated three times for reproducibility (*n* = 3).

### Effect of ML216 on DNA-damaged cells.

To check the effect of ML216 on DNA-damaged cells, the cultures were divided into two equal parts. One part was treated with 0.005% MMS for 2 h, and MMS was washed off after treatment. The cells were allowed to grow in complete medium containing various concentrations of ML216 (0.1 nM to 1,000 μM) for one generation (48 h). The second aliquot of the culture was maintained in the presence of ML216 without MMS treatment. In the positive control, MMS and ML216 were not added. Each assay was repeated three times for reproducibility (*n* = 3).

### PCR-based method to quantify DNA damage.

An amplification-based technique was performed to measure nuclear DNA damage as previously described, with modifications (32). Briefly, ring-stage parasites were pretreated with sublethal doses of the drug atovaquone (0.3 nM), ML216 (1 μM), or B02 (1 μM), followed by exposure to UV light (100 J/m^2^) at the trophozoite stage to induce DNA damage. After treatment, the mock-treated or the drug-treated cultures were grown in the presence of the respective drug-containing media for 48 h. Genomic DNA was collected before (untreated sample) and immediately after (0-h sample) UV treatment and every 12 h until 48 h. The isolated genomic DNA was quantified using a SYBR green I dye-based standard plot method. An equal amount of genomic DNA was taken to set up PCR for amplifying long- and short-range fragments (7,200 bp and 269 bp). Primer sets OMKB463 and -464 and OSB94 and -95 were used to amplify the long-range PCR and short-range PCR products, respectively. The PCR products were quantified using SYBR green I dye, and the fluorescence readings of the long-range PCR product were normalized using the readings of the short-range product. The amount of damaged DNA at any given time point was deduced from the following equation: damaged DNA = 1 − (fluorescence intensity of the long PCR product/fluorescence intensity of the short PCR product × 26.76), where the factor 26.76 represents the ratio of the long-range PCR amplicon size to the short-range PCR amplicon size. The amounts of damaged DNA from the untreated sample and the 0-h sample were considered 0 and 100%, respectively. The amount of residual damage at each time point was plotted using GraphPad Prism software. The *P* value was calculated using the two-tailed *t* test (* means a *P* value of <0.05, ** means a *P* value of <0.01, *** means a *P* value of <0.001, and N.S. means not significant).

### Fixed-ratio method to determine the interaction between ML216 and DHA/CQ.

Synchronous P. falciparum cultures of the trophozoite stage were used to determine the interactions between ML216 and DHA/CQ/ATQ. A fixed-ratio method was performed as previously mentioned (33). A Giemsa stain-based evaluation of parasitemia was done to estimate percent inhibition. A semilog graph was plotted for each combination to determine the IC_50_ value using GraphPad Prism software. The fractional inhibitory concentration (FIC) for each drug was determined by using the equation FIC = IC_50_ of the drug in the mixture/IC_50_ of the drug alone. The FIC values of both drugs were used to determine the interaction between ML216 and DHA by using the following equation: ΣFIC = (IC_50_ of DHA in the mixture/IC_50_ of DHA alone) + (IC_50_ of ML216 in the mixture/IC_50_ of ML216 alone). An isobologram was plotted by using GraphPad Prism software. An ΣFIC of <1 represents synergism, ΣFICs of ≥1 and 2 represent an additive interaction, and an ΣFIC of ≥2 represents antagonism. A similar equation was used for the CQ/ATQ and ML216 combination.
