# Supplementary figures and images for: Elucidation of DNA Repair Function of PfBlm and Potentiation of Artemisinin Action by a Small-Molecule Inhibitor of RecQ Helicase
Source: mSphere. 2020 Nov 25;5(6):e00956-20. doi: 10.1128/mSphere.00956-20 (PMC7690958; doi:10.1128/mSphere.00956-20)

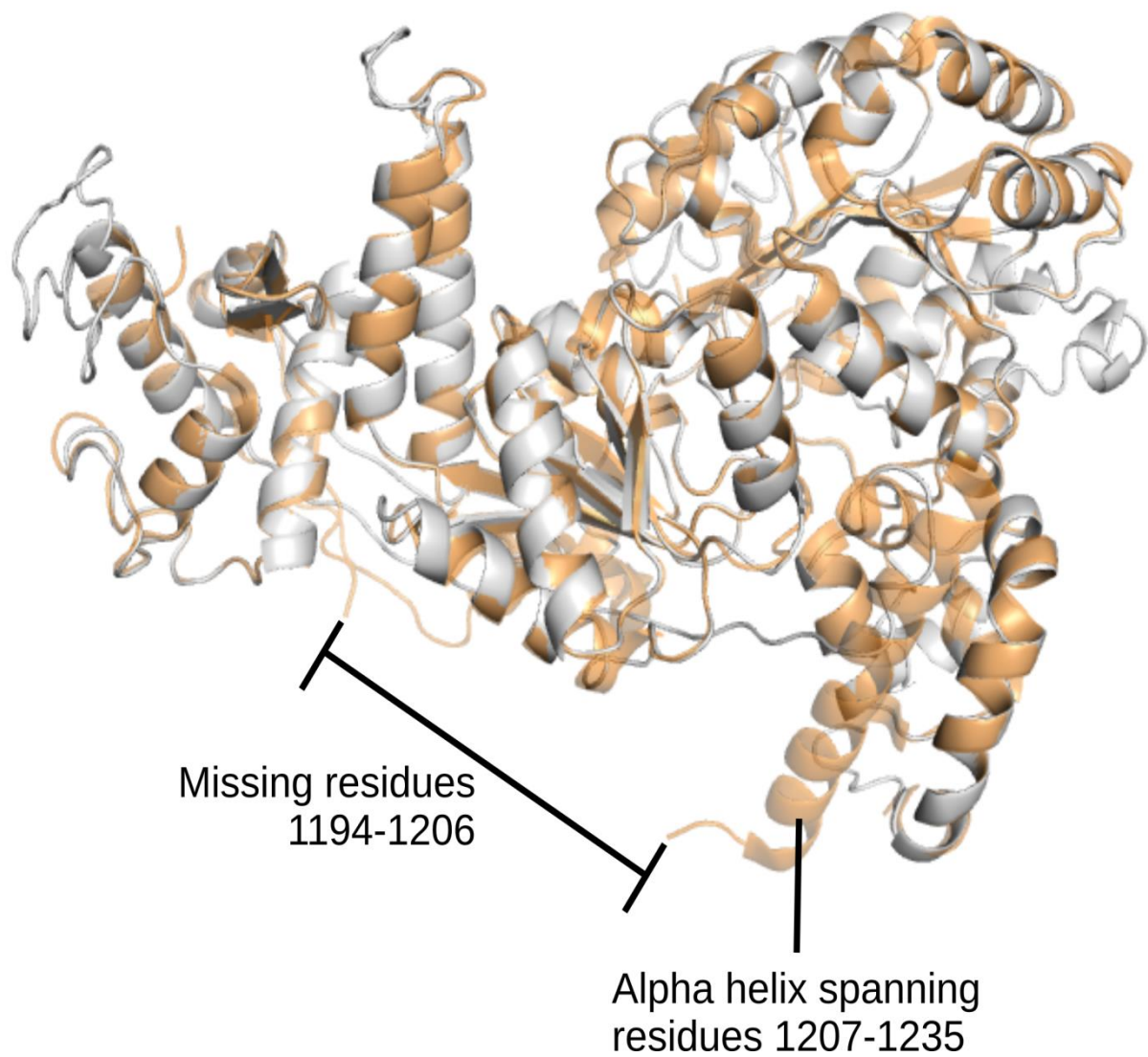

Supplement: FIG S1 [file mSphere.00956-20-sf001.pdf]

**A.** $IC_{50} = 6.31 \mu M$  (3D7)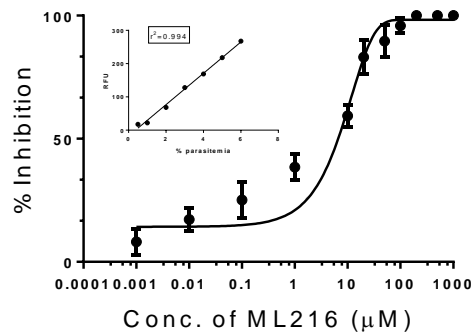**B.** $IC_{50} = 3.24 \mu M$  (Dd2)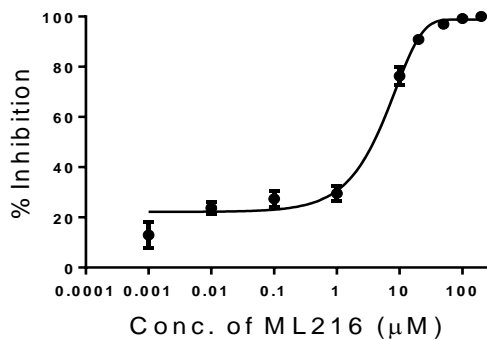**C.** $IC_{50} = 2.19 \mu M$  (PfK13R539T)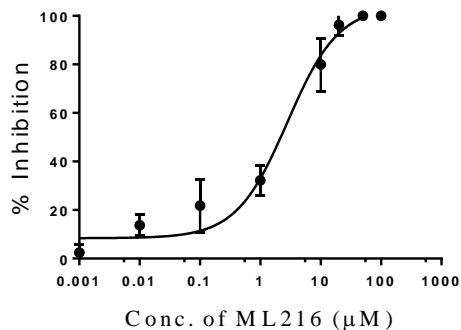**D.** $IC_{50} = 55.2 \mu M$  (3D7)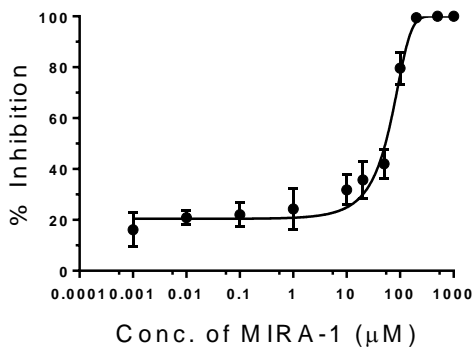**E.** $IC_{50} = 58 \mu M$  (Dd2)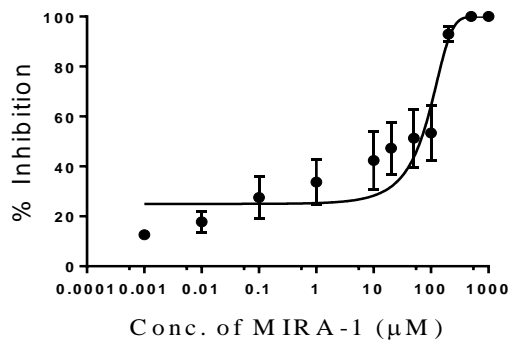

Supplement: FIG S2 [file mSphere.00956-20-sf002.pdf]
