# Supplementary material for: Elucidation of DNA Repair Function of PfBlm and Potentiation of Artemisinin Action by a Small-Molecule Inhibitor of RecQ Helicase
Source: mSphere. 2020 Nov 25;5(6):e00956-20. doi: 10.1128/mSphere.00956-20 (PMC7690958; doi:10.1128/mSphere.00956-20)
Supplement: TABLE S1 [file mSphere.00956-20-st001.pdf]

Supplementary Table S1.

Free energies of binding for inhibitors bound to PfBlm

Inhibitor name and binding site	ΔE_{MM} (kcal/mol)	$\Delta G_{solv,\ polar}$ (kcal/mol)	$\Delta G_{solv,\ non\text{-}polar}$ (kcal/mol)	$\Delta G_{binding}$ (kcal/mol)
ML216 at ATP-binding site	-56.6	33.2	-4.5	-27.9
ML216 in DNA-binding region	-51.9	32.8	-3.7	-22.7
MIRA-1 at ATP-binding site	-36.7	27.0	-2.7	-12.4
