# Supplementary material for: Elucidation of DNA Repair Function of PfBlm and Potentiation of Artemisinin Action by a Small-Molecule Inhibitor of RecQ Helicase
Source: mSphere. 2020 Nov 25;5(6):e00956-20. doi: 10.1128/mSphere.00956-20 (PMC7690958; doi:10.1128/mSphere.00956-20)
Supplement: TABLE S2 [file mSphere.00956-20-st002.pdf]

Supplementary Table S2.

FIC values of drug combinations

Strain	DHA:ML216	FIC$_{DHA}$	FIC$_{ML216}$	∑FIC	CQ:ML216	FIC$_{CQ}$	FIC$_{ML216}$	∑FIC	ATQ:ML216	FIC$_{ATQ}$	FIC$_{ML216}$	∑FIC
3D7	5:0	1	0	1	5:0	1	0	1	5:0	1	0	1
	4:1	0.64	0.22	0.86	4:1	0.56	0.31	0.87	4:1	0.73	0.27	1
	3:2	0.52	0.48	1.0	3:2	0.34	0.50	0.84	3:2	0.54	0.55	1.09
	2:1	0.27	0.64	0.91	2:1	0.17	0.60	0.77	2:1	0.37	0.87	1.24
	1:4	0.08	0.48	0.56	1:2	0.09	0.63	0.72	1:2	0.18	0.98	1.16
	0:5	0	1	1	0:5	0	1	1	0:5	0	1	1
Dd2	5:0	1.0	0	1	5:0	1.0	0	1	5:0	1	0	1
	4:1	0.23	0.13	0.36	4:1	0.28	0.12	0.40	4:1	0.85	0.17	1.02
	3:2	0.40	0.29	0.69	3:2	0.21	0.16	0.37	3:2	0.63	0.34	0.97
	2:1	0.25	0.42	0.67	2:1	0.20	0.34	0.54	2:1	0.52	0.54	1.06
	1:4	0.25	0.64	0.89	1:4	0.03	0.21	0.24	1:4	0.28	0.88	1.16
	0:5	0	1	1	0:5	0	1	1	0:5	0	1	1
PfK13R539T	5:0	1	0	1	n.d.	n.d.	n.d.	n.d.	n.d.	n.d.	n.d.	n.d.
	4:1	0.5	0.08	0.58	n.d.	n.d.	n.d.	n.d.	n.d.	n.d.	n.d.	n.d.
	3:2	0.55	0.25	0.8	n.d.	n.d.	n.d.	n.d.	n.d.	n.d.	n.d.	n.d.
	2:1	0.26	0.29	0.55	n.d.	n.d.	n.d.	n.d.	n.d.	n.d.	n.d.	n.d.
	1:4	0.11	0.32	0.43	n.d.	n.d.	n.d.	n.d.	n.d.	n.d.	n.d.	n.d.
	0:5	0	1	1	n.d.	n.d.	n.d.	n.d.	n.d.	n.d.	n.d.	n.d.

n.d.: not determined
