# Supplementary material for: Elucidation of DNA Repair Function of PfBlm and Potentiation of Artemisinin Action by a Small-Molecule Inhibitor of RecQ Helicase
Source: mSphere. 2020 Nov 25;5(6):e00956-20. doi: 10.1128/mSphere.00956-20 (PMC7690958; doi:10.1128/mSphere.00956-20)
Supplement: TABLE S3 [file mSphere.00956-20-st003.pdf]

**Supplementary Table S3.**

Primers used in this study

| Primer | Primer Sequence | Purpose |
|---|---|---|
| OMKB332 | ATTCGAGATAAAGGAATTATTGAAG | F.P. to amplify *PfBLM* for Semi quantitative PCR |
| OMKB333 | ATGCTTCCTCTTTACATTCATAG | R.P. to amplify *PfBLM* for Semi quantitative PCR |
| OSB94 | CTGTAACACATAATAGATCCGAC | F.P. to amplify *PfARP* for Semi quantitative PCR and short-range PCR product |
| OSB95 | TTAACCATCGTTATCATCATTATTTC | F.P. to amplify *PfARP* for Semi quantitative PCR and long-range PCR product |
| OMKB394 | TCAGGATCCATGGTGACGAAGCCGTCAC | F.P. to amplify *ScSGS1* for cloning in pBFM vector |
| OMKB395 | TCAGTCGACCTTTCTTCCTCTGTAGTGAC | R.P. to amplify *ScSGS1* for cloning in pBFM vector |
| OMKB372 | TCAGGATCCATGAATGAAGATGCTATGAAAATTTTG | F.P. to amplify *PfBLM* for cloning in pBFM, pGEX-6P2 and pGADC1 vectors |
| OMKB373 | TCAGTCGACAATTTTCCTTGGAATTTTAAATGAAG | R.P. to amplify *PfBLM* for cloning in pBFM vector |
| OMBK382 | CTAGTCGACTCAAATTTTCCTTGGAATTTTAAATG | R.P. to amplify *PfBLM* for cloning in pGEX-6P2 and pGADC1 vectors |
| OMKB19 | ATCGGATCCATGAAACAAGCAAATACAAAAG | F.P. to amplify *PfRAD51* for cloning in pGBDUC-1 vector |
| OMKB17 | ATCGTCGACTTTATTTTTCCTCATAATCTGC | R.P. to amplify *PfRAD51* for cloning in pGBDUC-1 vector |
| OMKB610 | ATCGGTACCATGAATGAAGATGCTATGAAAATTTTG | F.P. to amplify *PfBLM* for cloning in pARL vector |
| OMKB611 | ATCCCTAGGAATTTTCCTTGGAATTTTAAATGAAG | R.P. to amplify *PfBLM* for cloning in pARL vector |
| OMKB666 | AAATTAAGGATCTCCCACCACCTG | Overlapping primer to create *PfblmK83R* mutation |
| OMKB667 | CAACAGGTGGTGGGAGATCC | Overlapping primer to create *PfblmK83R* mutation |
| OMKB276 | AAGAATTGGGACAACTCC | R.P. to amplify GFP for confirming Transfection |
| OMKB463 | TCAGTCGACATGTTGAATGATATGAATGATAAAAAAG | F.P. to amplify long-range PCR product |
| OMKB464 | TCAGTCGACTCAACCTATGTAACCTTTACACTTC | R.P. to amplify long-range PCR product |

F.P: Forward primer, R.P: Reverse primer
