# Supplementary material for: Elucidation of DNA Repair Function of PfBlm and Potentiation of Artemisinin Action by a Small-Molecule Inhibitor of RecQ Helicase
Source: mSphere. 2020 Nov 25;5(6):e00956-20. doi: 10.1128/mSphere.00956-20 (PMC7690958; doi:10.1128/mSphere.00956-20)
Supplement: TABLE S4 [file mSphere.00956-20-st004.pdf]

Supplementary Table S4.

List of yeast strains used in this study

Strain name	Genotype	Source
PJ69-4A	*MATa trpl-901 leu2-3,112 ura3-52 his3-200 ga14Δ ga180Δ LYS2::GALl-HIS3 GAL2-ADE2 met2::GAL7-lacZ*	(1)
SNY1	*MATα leu2-3,112 trp1-1 can1-100 ura3-1 ade2-1 his3-11,15, sgs1Δ::KAN^r*	This study
SNY2	*MATα leu2-3,112 trp1-1 can1-100 ura3-1 ade2-1 his3-11,15, sgs1Δ::KAN^r pBFM / ScSGS1*	This study
SNY3	*MATα leu2-3,112 trp1-1 can1-100 ura3-1 ade2-1 his3-11,15, sgs1Δ::KAN^r pBFM / PfBLM*	This study
SNY4	*MATα leu2-3,112 trp1-1 can1-100 ura3-1 ade2-1 his3-11,15, sgs1Δ::KAN^r pBFM / PfblmK83R*	This study
SNY5	*MATα leu2-3,112 trp1-1 can1-100 ura3-1 ade2-1 his3-11,15, sgs1Δ::KAN^r pBFM / PfWRN*	This study
SNY6	*MATα leu2-3,112 trp1-1 can1-100 ura3-1 ade2-1 his3-11,15, sgs1Δ::KAN^r pBFM*	This study
SNY7	*MATa trpl-901 leu2-3,112 ura3-52 his3-200 ga14Δ ga180Δ LYS2::GALl-HIS3 GAL2-ADE2 met2::GAL7-lacZ pGADC1/PfBLM*	This study
SNY8	*MATa trpl-901 leu2-3,112 ura3-52 his3-200 ga14Δ ga180Δ LYS2::GALl-HIS3 GAL2-ADE2 met2::GAL7-lacZ pGADC1/PfBLM pGBDUC1/PfRAD51*	This study
SNY9	*MATa trpl-901 leu2-3,112 ura3-52 his3-200 ga14Δ ga180Δ LYS2::GALl-HIS3 GAL2-ADE2 met2::GAL7-lacZ pGADC1/PfBLM pGBDUC1*	This study
SNY10	*MATa trpl-901 leu2-3,112 ura3-52 his3-200 ga14Δ ga180Δ LYS2::GALl-HIS3 GAL2-ADE2 met2::GAL7-lacZ pGBDUC1/PfRAD51*	This study
SNY11	*MATa trpl-901 leu2-3,112 ura3-52 his3-200 ga14Δ ga180Δ LYS2::GALl-HIS3 GAL2-ADE2 met2::GAL7-lacZ pGBDUC1/PfRAD51 pGADC1*	This study
SNY12	*MATa trpl-901 leu2-3,112 ura3-52 his3-200 ga14Δ ga180Δ LYS2::GALl-HIS3 GAL2-ADE2 met2::GAL7-lacZ pGBDUC1 pGADC1*	This study
SNY13	*MATa trpl-901 leu2-3,112 ura3-52 his3-200 ga14Δ ga180Δ LYS2::GALl-HIS3 GAL2-ADE2 met2::GAL7-lacZ pGBDUC1/PfalMRE11 pGADC1/PfBLM*	This study
SNY14	*MATa trpl-901 leu2-3,112 ura3-52 his3-200 ga14Δ ga180Δ LYS2::GALl-HIS3 GAL2-ADE2 met2::GAL7-lacZ pGBDUC1/PfalMRE11*	This study
SNY15	*MATa trpl-901 leu2-3,112 ura3-52 his3-200 ga14Δ ga180Δ LYS2::GALl-HIS3 GAL2-ADE2 met2::GAL7-lacZ pGBDUC1/PfalMRE11 pGADC1*	This study
SNY16	*MATa trpl-901 leu2-3,112 ura3-52 his3-200 ga14Δ ga180Δ LYS2::GALl-HIS3 GAL2-ADE2 met2::GAL7-lacZ pGBDUC1*	This study
SNY17	*MATa trpl-901 leu2-3,112 ura3-52 his3-200 ga14Δ ga180Δ LYS2::GALl-HIS3 GAL2-ADE2 met2::GAL7-lacZ pAGDC1*	This study

Reference:

1. Badugu SB, Nabi SA, Vaidyam P, Laskar S, Bhattacharyya S, Bhattacharyya MK. 2015. Identification of Plasmodium falciparum DNA Repair Protein Mre11 with an Evolutionarily Conserved Nuclease Function. PLoS One 10:e0125358.
